# Electrical Tumor Detection Probe Calibrated to Diagnose Gastrointestinal Cancer Mass in Real-Time

**DOI:** 10.3390/jcm13195823

**Published:** 2024-09-29

**Authors:** Narges Yousefpour, Habibollah Mahmoodzadeh, Reihane Mahdavi, Mohammad Reza Fattahi, Amirmohsen Jalaeefar, Hossein Ataee, Fereshteh Ameli, Farzane Hajighasemi, Hadi Mokhtari Dowlatabad, Sepideh Mansouri, Omid Nabavian, Seyed Rouhollah Miri, Mohammad Abdolahad

**Affiliations:** 1 Nano Bioelectronics Devices Lab, Cancer Electronics Research Group, School of Electrical and Computer Engineering, Faculty of Engineering, University of Tehran, Tehran 1439957131, Iran; narges.yousefpour@ut.ac.ir (N.Y.); reihane.mahdavi@ut.ac.ir (R.M.); hataee@ut.ac.ir (H.A.); f.hajighasemi@ut.ac.ir (F.H.); mokhtari.hadi@ut.ac.ir (H.M.D.); 2 Cancer Institute, Imam Khomeini Hospital, Tehran University of Medical Sciences, Tehran 1419733141, Iran; hmahmoodzadeh@tums.ac.ir (H.M.); mrfatahi93@gmail.com (M.R.F.); jalaeefar@gmail.com (A.J.); fereshtehameli@gmail.com (F.A.); sepide.mansurii@gmail.com (S.M.); o_nabavian@sina.tums.sc.ir (O.N.); 3 School of Advanced Technologies in Medicine, Shahid Beheshti University of Medical Sciences, Tehran 1968917313, Iran; 4 UT&TUMS Cancer Electronics Research Center, University of Tehran, Tehran 1417935840, Iran

**Keywords:** gastrointestinal cancer, intraoperative, classification, real-time detection, Impedance phase slope, electrical Impedance spectroscopy

## Abstract

**Background**: The primary objective of this research is to propose an intra-operative tumor detection probe calibrated on human models of gastrointestinal (G.I.) cancers, enabling real-time scanning of dissected masses. **Methods**: Electrical Gastrointestinal Cancer Detection (EGCD) measures impedimetric characteristics of G.I. masses using a handpiece probe and a needle-based head probe. Impedance Phase Slope (IPS) and impedance magnitude (Z1kHz) are extracted as the classification parameters. EGCD was tested on palpable G.I. masses and compared to histopathology results. **Results**: Calibration was carried out on 120 GI mass samples. Considering pathological results as the gold standard, most cancer masses showed Z1kHz between 100 Ω and 2500 Ω while their IPS was between −15 and −1. The EGCD total sensitivity and specificity of this categorization in G.I. cancer patients with palpable tumors were 86.4% and 74.4%, respectively (*p*-value < 0.01). **Conclusion**: EGCD scoring can be used for 3D scanning of palpable tumors in G.I. tumors during surgery, which can help clarify the tumors’ pathological response to neoadjuvant chemotherapy or the nature of intra-operative newly found G.I. tumors for the surgeon to manage their surgical procedure better.

## 1. Introduction

Gastric cancer is a prevalent type of cancer that is responsible for a significant number of cancer-related deaths. According to recently released WHO reports, while the incident rate of gastric cancer has decreased during the last decades, it still caused 7.7% of all cancer deaths and 5.6% of all cancer cases, about 75% of all new patients [1,2].

The incidence and mortality of GIC are variable due to the subtype of the GIC, geographical location, age, and gender of the patients worldwide [3].

Gastroesophageal cancers (GOCs) include oesophageal and gastric cancers (G.C. and O.C.). They have one of the highest incidence rates of all cancers and cause a drastic ratio of cancer-based mortality [4]. Despite new therapies, O.C. has a poor prognosis, with a five-year survival rate of less than 20% [5]. Patients with early stage G.C. have a 70–95% survival rate, while those with advanced disease have a 20–30% survival rate [6].

Esophageal cancer is an aggressive gastrointestinal malignancy with a five-year survival rate ranging from 15 to 25% worldwide. It is the sixth leading cause of cancer-related death, with poor prognosis and increasing incidence [7,8]. Patients diagnosed with oesophageal cancer tend to have poor results due to the high likelihood of metastases, even when the tumors are superficial. Although neoadjuvant therapies such as presurgical chemotherapy or chemoradiation have been effective in roughly 50% of patients, treating oesophageal carcinoma remains a significant challenge. A multidisciplinary approach is necessary to tackle this disease. While considerable progress has been made in the curative approach to oesophageal cancer, challenges remain. Oesophageal cancer has few targetable mutations, and highly heterogeneous tumors have a high mutational frequency that makes it challenging to set appropriate diagnostic and curative approaches. Cost is also an issue, as well as affordable and efficient high-throughput sequencing with a straightforward clinical application, whether in stratifying prognosis or tailoring therapies [9,10].

The primary treatment for early gastric cancer is endoscopic resection; in contrast, non-early operable gastric cancer is treated with surgery, and advanced gastric cancer is treated with sequential lines of chemotherapy. Treatment with chemotherapy before surgery increases the chance for curative resection, eliminates early microscopic spread, and allows an in-vivo response assessment of therapy [11]. On the other hand, while randomized trials of postoperative chemotherapy have been conducted, they have not found conclusive evidence that adjuvant chemotherapy improves the survival rate of patients who have undergone surgery. None of the trials or meta-analyses have factored in the extra time that chemoradiotherapy adds to the patient’s recovery. For instance, if chemoradiation provides a survival advantage of a few months but also requires a few months of additional recovery time compared to surgery alone, then any benefit gained would be nullified [12].

It is important to note that many patients with gastric cancer may not be diagnosed until the advanced stage, which leads to poor prognosis after surgical resection. However, in recent years, patients with locally advanced gastric cancer have gradually undergone perioperative comprehensive treatment. Clinicians use imaging approaches to assess the treatment response of tumors. Observing the volume reduction of tumor lesions in the early therapy period is challenging and cannot be an excellent indicator for monitoring treatment response [13].

The current preoperative imaging techniques may not accurately evaluate the complete response in neoadjuvant G.I. cancers. New methods for precisely distinguishing tumors and fibrosis during surgery can overcome the limitations and hazards of preoperative staging. One way to achieve this is by identifying the pathological characteristics of tumor lesions. Identifying the pathological characteristics of tumors involves accurately assessing the response to chemo/radiation therapy in neoadjuvant cases and diagnosing cancer in surgically removed masses to reduce the risk of recurrence and avoid the need for postoperative adjuvant treatment.

Besides the surgical procedure recommended by the surgeon (e.g., APR, LAR, Colectomy, etc.), a complete pathological evaluation of the whole dissected tumor (which may be entangled by normal tissues) is vital for a comprehensive assessment of the procedure. However, this evaluation requires subsequent sectioning (up to 80 cuts in some samples), which can be time-consuming and may induce bias in non-expert pathologists when they do not find any malignancies in the first 50% of the H&E slides. Sometimes, just one slide among tens presents a trace of malignant cells, which can change the patient’s future therapeutic trend [14].

New activities were attempted to find a real-time method with pathological evaluations to complete scanning of the whole tissue about malignancy [15,16]. Recently, an electrical impedance-based tumor detection spectroscopy was developed and applied to characterize human palpable breast lesions to distinct high-risk from benign masses. With the help of two electrical parameters, Impedance Phase Slope (IPS) in the frequency ranges of 100 kHz to 500 kHz and impedance magnitude in f = 1 kHz (Z1kHz), which had been previously calibrated by breast and thyroid pathology [17,18,19]

This study presents an impedance-based needle-shaped sensor to evaluate all suspicious masses in superficial and deep G.I. lumens lesions and detect remaining cancer or high-risk excision-required tumors. This verification is a new application to assess G.I. freshly dissected masses. The Electrical Gastrointestinal Cancer Detection (EGCD) system was employed in Gastrointestinal Cancer surgeries to evaluate its calibration effectiveness in real-time scanning of tumor malignancy. Calibration was carried out on 122 G.I. mass samples (Esophagus, Gastric, Rectum, and Colon) from neoadjuvant and adjuvant cases due to the histopathological gold standard of the tested location. The study was a proof of concept with no perturbations in the trend of diagnosis and therapy. Only observational signal recording was conducted, and the histopathology of the specimen blindly evaluated the EGCD calibration scoring. This system not only helps the surgeon to be informed about the presence of tumors in the surgical field of neoadjuvant cases (which may be an incomplete response to chemotherapy and further consideration of surgeon during the surgery is required) but also may help pathologists to identify the most suspicious regions, especially in neo-adjuvant cases, and cover all the deficiencies in detecting an incomplete response of G.I. cancers. This modality can also aid in preoperative staging, ensuring patients receive the best possible care.

## 2. Materials and Methods

### 2.1. EGCD Structure and Measurement Protocols

**Figure 1:**
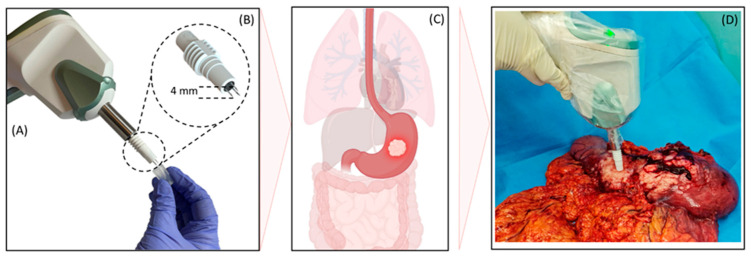
The EGCD’s read-out system and function. (**A**) EGCD handpiece. (**B**) An enlarged view of the head probe illustrates the needle dispersion’s associated dimension. (**C**) Structure of tumoral gastric. (**D**) EGCD applies to the tumoral gastric, where the tumor invades the outer lining.

EGCD is a real-time Electrical Impedance Spectroscopy (EIS) system with a needle-based probe to diagnose palpable gastrointestinal tract tumors intraoperatively. The EGCD comprises a handpiece probe (Figure 1A) and a head probe (Figure 1B). The head probe includes three stainless steel needles (G24) and measures the conformation triangular distance of 3 mm from each other and the length of 15 mm. A plastic shield covers the needles, and they are electrically isolated from each other. 2 mm from the tip of the needles are uncovered and exposed to the tissue. A cap on the head probe’s needle allows tissue penetration between 6 and 15 mm. Only 6 mm of the needle enters the tissue while the cap is on the head probe, which is how this cap works. The head probe’s cap can be removed and inserted into the tumor to a depth of 15 mm to reach deeper tumors that are more challenging to sense.

Due to stimulations, the needle’s electrically active site and sensing depth is 0.5 mm. Still, the sensor can enter a mass up to a depth of 4 mm and volumetrically scan the entire mass without causing tissue damage.

The electronic read-out board of the system embedded in the EGCD handpiece probe is a customized precision impedance meter that works in constant voltage mode which is designed and manufacture in Tehran, Iran. The impedance spectroscopy is accomplished by applying an alternative voltage (0.4 V amplitude and frequency of 1 Hz to 500 kHz) across the three needle electrodes (two of them are at the same potential to broaden the measurement area). This system stimulates the tissue with an electric field of about 200 v/m. Then, the electrical current signal is measured, impedance magnitude and phase are calculated, and classification parameters are extracted. Classification features extracted from measurements are Z1kHz (impedance magnitude in f = 1 kHz) and IPS (Impedance Phase Slope in the frequency ranges of 100 kHz–500 kHz). EGCD then reports tumor status with “Negative” and “Positive” for each measurement as the classification result.

### 2.2. Test Methodology

The surgeon employs the EGCD probe into many regions of all G.I. cancer palpable dissected tumors immediately after dissection and before the conventional fixation processes under the pathologist’s supervision who was present in the surgery room to avoid possible conflicts with routine pathology procedures of the sample; hence, no perturbation was induced in standard sample sectioning, preparation, and staining. The surgical plan such as Esophagectomy, total/subtotal/partial Gastrectomy, Colectomy, Abdominal Perineal Resection (APR), Very Low Anterior Resection (VLAR), and Low Anterior Resection (LAR), re-operation due to local re-currency or distant tumor (with the chance of curative or palliative surgeries), and finally, second surgery due to involved margins (in whom the re-surgery is required) assessed for eligibility. Moreover, palpable lesions may include tumors undergoing chemo/radiation therapy or accidentally finding masses during the surgery.

After the surgeon assessed the dissected mass and based on the patient’s presurgical evaluation (TNM staging), the EGCD was applied to the tumor (Figure 1C). In some cases, such as patients with tumors in the stages of T3 and T4, where cancer has spread through the outer lining and with the possibility of nearby tissue and organ involvement, the tumor is accessible and visible through the outer surface. In these patients with accessible tumors through the outer lining, there is no need to open the specimen to reach the mucosal surface (Figure 1D). In other cases, in which the tumor is inaccessible from the outer lining, the pathologist opens the specimen to expose the mucosal surface if the surgeon deems it necessary to conduct additional investigations. This step involves cutting through the wall of the luminal tissue from one end of the sample to the other and trying not to distort the appearance of the tumor and disrupt the tumor’s correlation with underlying structures. Once the specimen is open, the surgeon/pathologist observes the appearance and thickness of the wall. If strictures are present, the surgeon/pathologist applies the EGCD probe on the palpable suspicious lesion of the dissected mass between three to ten times to record the impedance magnitude response and phase for feature extraction and classification of the EGCD.

To accurately evaluate gastrointestinal cancers, due to their highly varied molecular and physical characteristics, the EGCD probe should be inserted into each suspicious palpable lesion between three to ten times, depending on the size of the tumor or the extent of the tumoral area. In the following, these evaluated lesions were marked by the pathologist. Each sample was individually reported as an EGCD sample for further validation (in addition to a conclusive diagnosis of the patient’s dissected mass). All the samples underwent conventional pathological H&E procedures as the gold standard. Immunohistochemistry (IHC) was also performed upon the pathologist’s request. The EGCD response of the tumor was then compared with the pathological diagnosis of each sample as the gold standard. Based on the pathology diagnosis of the samples, they were marked as True or False by the EGCD system. So, we established a calibration for G.I. tumor detection to assist the surgeon during adjuvant or neoadjuvant G.I. cancer surgeries.

The EGCD head probe was designed as a disposable sensor using medical-grade needles (G24) to prevent the seeding of residues that may be stuck to the needle from one site to another.

### 2.3. EGCD Calibration in Human Samples

We designed an observation clinical trial study to achieve the calibration for the EGCD probe in G.I. cancer detection as a surgeon/pathologist assistant. In this regard, we used a needle-based probe to scan the dissected masses from G.I. cancer patients in adjuvant or neoadjuvant states in palliative or curative surgical procedures.

### 2.4. Case Selection for Calibrating the Device

**Figure 2:**
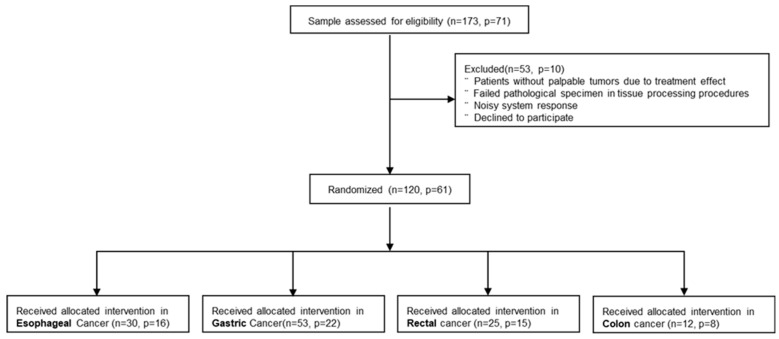
The study inclusion and exclusion in the cohort study.

One hundred thirty-one patients with G.I. cancers who underwent surgery in the Cancer Institute of Imam Khomeini Complex Hospital (from December 2021 to October 2022) were included in the present study, but finally, 61 were assigned. The inclusion criteria are the existence of presurgical radiological (MRI, CT scan, EUS, Colonoscopy, Sonography) and presurgical pathological evaluation results (colonoscopic/endoscopic biopsy) and having palpable G.I. tumors. Among 61 gastrointestinal cancer patients, 10 were excluded because of non-palpable tumors after chemo/radiotherapy, failed pathological specimens in tissue processing procedures, noisy system response, and declining participation. Figure 2 represents the details of the inclusion and exclusion criteria.

### 2.5. Statistical Analysis

To assess the difference in classification criteria presented in this study, we used statistical analysis to compare the classification criteria with the pathological diagnosis of the EGCD system for all G.I. samples taken from patients. We calculated the *p*-value of chi-square with a confidence interval of 95% using IBM SPSS Statistics for Windows version 25. This helped us determine the statistical significance of the relationship between the classification criteria and the EGCD system. The chi-square test assessed whether there was a significant relationship between categorical variables.

Additionally, we utilized the Receiver Operating Characteristic (ROC) curve and Area Under the Curve (AUC) to demonstrate the sensitivity vs. specificity and efficacy of EGCD. Sensitivity measures the ability of the classification criteria to correctly identify true positives, while specificity measures the ability to identify true negatives correctly. The AUC quantifies the overall discriminatory power of the classification criteria. An AUC value close to 1 indicates excellent performance. The ROC curve visually portrays the trade-off between sensitivity (true positive rate) and specificity (true negative rate) across various classification thresholds.

All statistical analyses were conducted using IBM SPSS Statistics for Windows version, which is widely recognized for data analysis and hypothesis testing in research and clinical settings. Through these analyses, we evaluated the efficacy of the EGCD system in distinguishing between malignant and normal tissue.

### 2.6. Ethics

The project is performed according to the World Medical Association Ethics, Declaration of Helsinki, and the ethical principles and national standards for conducting Medical Research in Iran. All of the human tests were performed under the license of the Ethics Committee of Tehran University of Medical. Institutional review board (IRB) or research ethics committee (REC) approval and clinical trial registration numbers of the project is I.R.TUMS.VCR.REC.1397.532.

## 3. Results

Table 1 shows the demographic characteristics of the patients and tumors recruited in the present study. The study includes G.I. cancers (Adenocarcinoma: *n* = 52 (52.5%), Squamous Cell Carcinoma (SCC): *n* = 17 (27.9%), Neuroendocrine Carcinoma: *n* = 2 (3.3%), Leiomyosarcoma: *n* = 1 (1.6%), Moderate Epithelial Dysplasia: *n* = 1 (1.6%), Signet Ring Cell Carcinoma (SRCC): *n* = 2 (3.3%), Serous Carcinoma: *n* = 2 (3.3%), Mixed Adeno-Neuro-Endocrine Carcinoma (MANEC): *n* = 1 (1.6%), and Tubulovillous Adenoma: *n* = 3 (4.9%). 48 (78.7%) patients underwent chemo/radiotherapy before surgery. Men and women comprised 52.5% and 47.5% of the eligible patients, respectively. The average age of the cohort study was 59. Tumor grade, presurgical imaging result, and treatment response of the tumors evaluated by pathology are included in Table 1.

According to Table 1, the *p*-value with a confidence interval of 0.95 determines that there was no significant relationship between the pathological status of samples and some parameters such as age (*p* = 0.383), sex (*p* = 0.264), and presurgical radiological scores were observed (*p* = 0.707). In contrast, a significant relationship was observed between the EGCD score and pathological involvement of G.I. tumor samples (*p* = 5.921 × 10^−7^ < 0.01).

**Figure 3:**
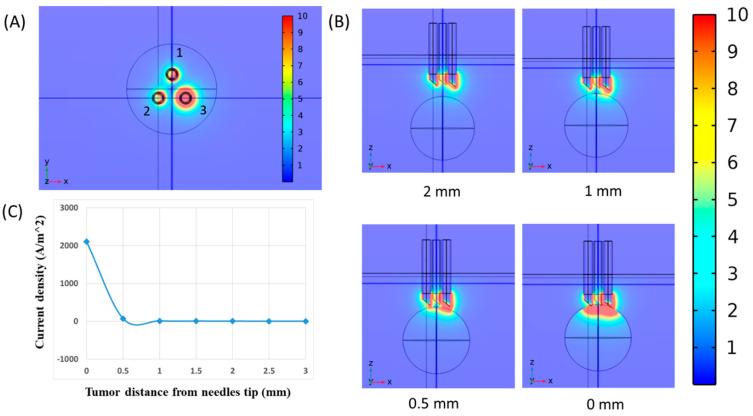
Simulation of normalized current density in EGCD for f = 1 KHz and configuration of longitudinal proximity of cancerous mass. (**A**) Representation of max electrical current density at electrode places. The 1-3 is top view of the electrodes configuration. It is worth mentioning that the electrodes named 1 and 2 have the same potential to increase the measurement area. The simulation showed that the tip of the needles is the most sensitive electrode of the head probe. (**B**) Simulation of electrical current penetration depth into the cancerous tumor in the different distances of the tumor from the tips of needles. (**C**) A two-dimensional diagram representing tumor distance from the tip of the needle on the *x*-axis and current density on the *y*-axis for all different distances of tumor from the tips of needles defines that the transition point of the EGCD head probe is about 0.5 mm from the needle tip.

Based on our previous experience with the impedimetric nature of carcinoma tumors, we designed an EGCD probe to apply to GIC samples. A multi-physics simulation of the EGCD probe was performed to estimate the electrical current penetration depth into the test sample and the effective measurement area. According to the current density in longitudinal proximity of cancerous mass (Figure 3A), the most sensitive area of the probe is about 0.5 mm around the tip of the needles. Simulation results in the frequency ranges of 1 Hz to 500 kHz in longitudinal configurations are provided (Figure 3B). It is measured by changing the tumor sphere lateral distance from the needle tip in some steps and measuring the critical point where a drastic change of about 98% has occurred in the electrical current density. This result shows that the needle should be completely inserted into the suspicious nodule for accurate measurement (Figure 3C).

**Figure 4:**
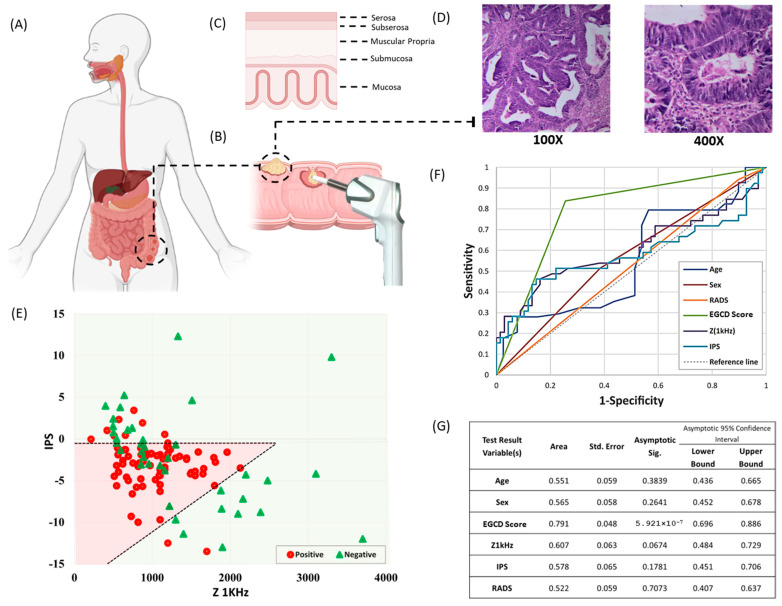
EGCD measurement, calibration, and scoring schematic at G.I. tract. (**A**) Structure of sizeable tumoral lesion in rectosigmoid junction. (**B**) The surgeon applied the EGCD probe to the dissected tumoral mass. (**C**) A magnified view of the layer structure of the mass that the EGCD encounters during measurement. (**D**) H&E assay of post-surgical pathology evaluation of the tumoral tissue. (**E**) A two-dimensional scattered diagram representing Z1kHz on the *x*-axis and IPS on the *y*-axis for all tested samples defines a primary calibration cut-off set. The green area is supposed to represent the negative region with the highest probability of benignity in G.I. samples. In contrast, the patterned red rectangle represents the positive region with the highest likelihood of malignancy in GIC samples. (**F**,**G**) Receiver operating characteristic (ROC) and comparison of AUC and *p*-value for IPS, Z1kHz, EGCD score, radiology score (RADS), age, and sex as the classification parameters.

EGCD was applied on freshly dissected GIC samples in the surgery room (Figure 4A) to check the range of two impedimetric parameters (Z1KHz and IPS) (Figure 4B) of malignant and benign/fibrotic lesions and also to find if precise results could be achieved for scoring or not. In this regard, it is evident that a precise comparison between the EGCD results and the microscopic structure and histopathology of the specimen is essential to evaluate the system’s accuracy (Figure 4C,D).

Two impedimetric characteristics were noted for each measured lesion of dissected GIC samples. All free and involved EGCD-evaluated samples were superimposed onto a two-dimensional graph with Z1KHz on the *x*-axis and IPS on the *y*-axis (the scored lesion became green if it was not involved in any malignancy and red if it contained cancer cells) (Figure 4E) The extreme range of Z1KHz was from 0 to 4000 ohm and IPS from −15 to 14. Subsequently, the cut-off values of classification of all samples visually selected according to the meaningful distribution of free and involved samples and their abundance from 61 GIC patients who underwent surgery, bordered as one Area in the Z1kHz-IPS diagram in which this Area was scored as an EGCD positive area. Samples out of this area were assumed to be an EGCD negative area. The EGCD-positive area is between two lines with a line equation of IPS = (64 × 10 ^(−4)^ × Z) − 17.56 and IPS = −1.

The EGCD total sensitivity, specificity, and accuracy of this categorization in GIC patients with palpable tumors are 86.4%, 74.4%, and 82.5%, respectively.

In this study, we used statistical analysis to compare the classification criteria with the pathological diagnosis of the EGCD system for all G.I. samples taken from patients. We calculated the *p*-value of chi-square with a confidence interval of 95% using IBM SPSS Statistics. This helped us determine the statistical significance of the relationship between the classification criteria and the EGCD system. The chi-square test assessed whether there was a significant relationship between categorical variables.

Additionally, we utilized the Receiver Operating Characteristic (ROC) curve and Area Under the Curve (AUC) to demonstrate the sensitivity vs. specificity and efficacy of EGCD. The AUC quantifies the overall discriminatory power of the classification criteria. An AUC value close to 1 indicates excellent performance. The ROC curve visually portrays the trade-off between sensitivity (true positive rate) and specificity (true negative rate) across various classification thresholds. Through these analyses, we evaluated the efficacy of the EGCD system in distinguishing between malignant and normal tissue.

The ROC curve analysis was carried out to check the reliability of EGCD in diagnosing G.I. tumors based on histopathology evaluation as the gold standard for dissected mass after the surgery. The Area under the curve (AUC) is 0.791 (Figure 4F), with a *p*-value < 0.01 (5.9214 × 10^−7^) for the EGCD score, which shows the excellent performance of EGCD criteria compared to other variables. At the same time, AUC for age, sex, and presurgical radiology evaluation was 0.621, 0.565, and 0.522, respectively (represented in Figure 4G). It is important to note that while the EGCD score is reasonably associated with permanent pathology, the Z1kHz and IPS values have AUCs of 0.607 and 0.578, respectively, and this indicates that these two impedimetric parameters cannot be used independently to determine EGCD. Z1kHz and IPS should be considered together to ensure a comprehensive and accurate diagnosis with high sensitivity and accuracy.

Another noteworthy point is the comparison of the Radiology Score (RADS) and EGCD. The AUC value for RADS is 0.522. Compared to the EGCD score, it is evident that the EGCD can effectively evaluate G.I. cancer and address the deficiencies of preoperative evaluation discussed in this article. This would be particularly beneficial for patients who have undergone preoperative treatment, as it can provide a more accurate evaluation of their condition, which is often lacking for surgeons in most cases. 

Due to this categorized calibration, 70 lesions were truly scored as positive (T.P.) and 29 as true negative (T.N.) samples. In contrast, 10 were falsely scored as positive (F.P.), and 11 were falsely scored as negative (F.N.). (Table 2). Sensitivity and specificity of EGCD evaluations were respectively about 81.3% and 71.4% for esophagus samples, 86.8% and 73.3% for Gastric samples, 100% and 100% for colon samples, and 81.3% and 77.8% for rectum samples based on post-surgical pathology evaluation as the gold standard.

A comparison was performed between presurgical imaging (CT-Scan and MRI) and EGCD score based on the pathology of dissected mass in both negative and positive samples (Table 2). Some cases were not truly clarified by imaging, while the EGCD truly scored the scanned mass in either the positive or negative category. The sensitivity and specificity of post-treatment imaging evaluation were 81.3% and 7.1% for esophagus samples, 100% and 20% for gastric samples, 100% and 0% for colon samples, and 93.3% and 11.1% for rectum samples, respectively. Compared to the EGCD sensitivity and specificity reported for those organs, EGCD performs better in interpreting and identifying negative samples.

The EGCD presented unique impacts as an observational tool based on the EGCD result compared to presurgical imaging. EGCD had good diagnostic accuracy compared to presurgical imaging for conclusive histopathological diagnosis. In this regard, among 54 patients with presurgical imaging suggesting the presence of the tumor, EGCD truly diagnosed the malignancy in 34 patients, and in 10 cases, the EGCD truly diagnosed patients with the absence of cancerous cells in their masses. In this regard, EGCD can help surgeons to better judge inflammatory fibrosis and malignant tumors near presurgical imaging reports.

**Figure 5:**
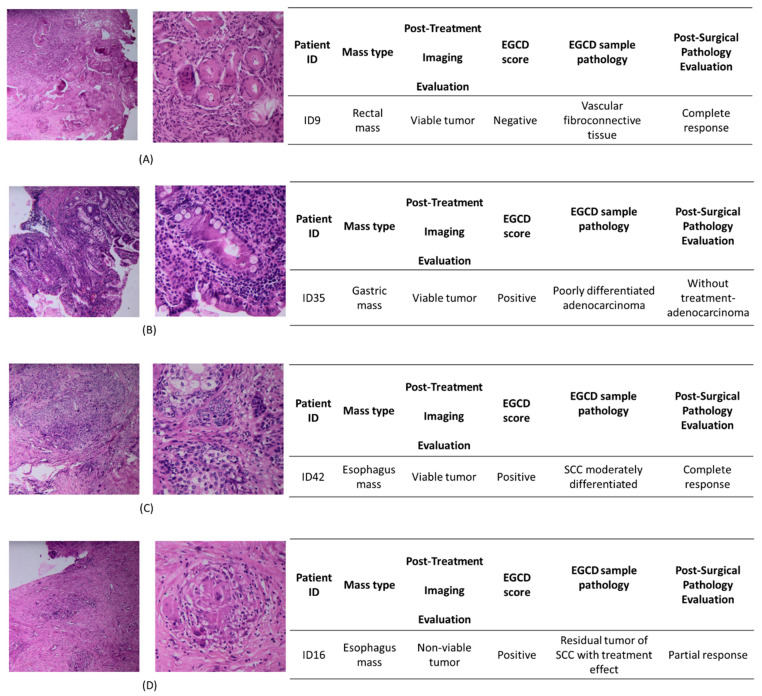
Picture of the H&E assay of G.I. mass and comparison of EGCD score, post-treatment imaging evaluation, and post-surgical pathology evaluation of each case. (**A**) The rectal mass is free of tumors. (**B**) Gastric mass involved by adenocarcinoma. (**C**) Esophagus mass involved by SCC. (**D**) Esophagus mass involved by SCC.

For instance, in the case of ID9, while presurgical imaging revealed the presence of cancer tumors, EGCD scored the lesions of that case as negative, which was confirmed by pathology (Figure 5A). In other cases, while just one lesion was involved by cancer, the EGCD truly found many more cancer-involved lesions (Figure 5B). Also, in some neoadjuvant cases in which primary permanent pathology of post-surgical samples indicated complete response of the organ to chemo/radiation therapy, more investigations via serial scoring from the locations resulted in a positive score by EGCD which was confirmed by the pathologist (the remaining SCC lesions in the esophagus). This is crucial in the patient’s post-surgical treatment regime (Figure 5C). It is worth mentioning that in some cases (patient ID16), the presurgical imaging revealed the complete response after chemo/radiation therapy for GIC patients, in which both EGCD evaluation during surgery and post-surgical pathology reports indicated that the patient has poor or no response to the treatment (Figure 5D).

## 4. Discussion

Electrical methods offer a new way to evaluate fresh cancer tumors in real-time during surgeries such as breast and thyroid cancer operations. One of the technologies developed for this purpose is impedance analysis. Among electrical tumor probing methods, Electrical Impedance Spectroscopy has its advantages, such as precision, simple handling, and wide and deep recorded data to be correlated with the functions and properties of biological tissues in normal and neoplastic states [17,18,19,20,21]. When biological tissues are subjected to alternating electrical excitation, they display a complex electrical impedance that depends on tissue composition, structure, health status, and physiological or pathological properties [22]. This means that normal and malignant tissues have different impedimetric parameters due to their distinct frequency-dependent dielectric relaxation and electric current-blocking abilities [23].

Bioimpedance analyzers (BIAs) are complementary devices for imaging techniques such as sonography. Their outcomes do not provide pathological indications and cannot be used for intraoperative tumor diagnosis.

Several reports have been published on impedance analysis of dissected human tumor masses; however, only a few researchers have focused on some gastrointestinal tumor analysis [21,24,25]. This limited focus may be due to the heterogeneous structure of G.I. tumors and the challenge of distinguishing between the dielectric behaviors of each layer, which respond differently to electrical stimulation. Such an analysis that provides calibration for real-time diagnosis of G.I. cancer could be beneficial and could assist in the intraoperative evaluation of tumors with reliable accuracy and sensitivity [26].

This project presents a new calibration for a pathologically classified methodology based on electrical impedance spectroscopy for real-time scanning of whole dissected G.I. tumors. This system would serve as a complementary tool for intraoperative diagnosis. The system was designed and built to assess all suspicious masses in superficial and deep G.I. lumen lesions and to detect any remaining cancer or high-risk excision-required tumors. In this article, we use our newly presented detection method [19] to establish calibration for G.I. cancers. This verification process represents a new application for assessing freshly dissected masses in the gastrointestinal region. The Electrical Gastrointestinal Cancer Detection (EGCD) system was utilized in gastrointestinal cancer surgeries to evaluate its effectiveness in calibrating real-time scanning of tumor malignancy.

The EGCD system approved that calibration areas with discriminated Z1KHz and IPS borders could be defined to score malignant lesions with experimentally achieved best sensitivity, specificity, and accuracy of 86.4%, 74.4%, and 82.5%.

The heterogeneous nature of G.I. tissues—a complex combination of epithelial, mucosal, serosal, fibrotic, and inflammatory cells among connective tissues and fibroblasts—could not lead to precise diagnostic results if we focused only on the impedance magnitude analysis. Therefore, the impedance magnitude results were combined with IPS results for accurate evaluation. The EGCD calibration found a new era with acceptable sensitivity and specificity in which the two slope lines separated the electrical response of involved lesions from free lesions while an area with a low occurrence value remained undiagnosable.

According to the data extrapolated from Figure 4E, most involved lesions showed IPS between −10 and −1 with the Z1KHz lower than 1500 Ω. The abundance of samples characterized by Z1KHz between 1500 and 2500 Ω with the same IPS (higher value in negative IPS region) becomes low. This phenomenon might be due to non-tumoral luminal/stromal tissues, which have higher impedance than completely tumor-involved tissues. However, it is worth noting that an individual region in the table was achieved as positively scored lesions with the sensitivity and specificity of 86.4% and 74.4%, respectively.

Such calibration clarified this heterogenicity and presented clinically acceptable scores to introduce distinct areas about electrical parameters of free or involved G.I. masses compared to conventional presurgical evaluations such as radiology. Among 14 cases with post-surgery pathology diagnosis of complete response to the neoadjuvant chemo/radiation therapy, our study, based on the achieved calibration, interestingly detected a trace of non-complete response in three patients. Also, it correctly declared pathologically complete responses in nine cases.

Although this approach shows the promising complete 3D evaluation of the whole dissected mass in real-time by the suggested figure of merits, some limitations should be addressed. Our results covered all types of G.I. cancers, and an individual trial for each kind of G.I. tumor may show more clarification in the sensitivity and specificity of EGCD in each subtype of G.I. cancer. We aimed to demonstrate the ability of EGCD in G.I. mass electrical scoring to overcome the non-correlative bioimpedance reports of previous papers on different G.I. masses [23,27,28,29,30]. Also, it is essential to note that when the mass completely disappears after chemoradiation therapy (non-palpable masses), the EGCD is not applicable for scoring because we cannot test all over a dissected G.I. tissue or organ without any palpable solid mass.

There are also some limitations that this study mentions regarding the application of ECGD to all different kinds of G.I. tumors, such as solid or ulcerated masses.

One of them is ischemia after dissection. Articles said that in dissected fresh tissue, the cellular structure changes with increasing ischemic times [28]. Additionally, such ischemia may be more significant in malignant lesions with high vascularity. In this study, based on the measurement procedure of the specimen explained above, samples were collected immediately after surgical dissection. If the specimen is tested with some delay, it can cause a loss of blood supply and ischemia that may affect the responses of the EGCD. However, in this trial, as the scoring was carried out immediately after dissection in the surgery room, the number of confirmed positive lesions in a more coherent area could be predictable.

The ECGD application must also consider other limitations in detecting G.I. tumors. As mentioned, tumor size and palpability matter due to the ECGD design. The EGCD’s head probe includes three stainless steel needles (G24) positioned 3 mm apart. There are 2 mm of the needles left uncovered and exposed to the tissue. All needles must be positioned inside the tumor tissue for valid evaluation. When voltage is applied to the needles, the current flows through them and into the tissue, allowing assessment of the tissue’s response. Accurate measurement of the electricity is essential. The heterogeneous nature of G.I. tissues could lead to imprecise results if only one examination result is relied upon. Therefore, EGCD should be applied to the lesion at least three times for accurate results. As a result, tumors smaller than 10 × 10 × 5 mm^3^ should not be evaluated with EGCD.

One of the specifications of this study is that the pathologist can prepare different slide sections from suspicious lesions, which EGCD positively scored for better diagnosing the neoadjuvant G.I. masses, especially in the esophagus and rectal cancer, due to the importance of responding to the neoadjuvant for the post-surgical treatment regime.

Additionally, the ROC curve showed lights for the EGCD application. A new form of score representation from our initial data in this paper must be discussed by the readers while it confirms non-correlative results reported from the impedance of intra-abdominal tumors in other articles.

As a result, 3D volumetric scanning near real-time scoring and the presented pathological calibration diagram may make the EGCD a promising device for G.I. tumor investigation. However, many more clinical trials are required to decide whether it is applicable.

The clinical consequences of applying EGCD may be a reduction in the number of slide sectioning (in positive cases, which guides the pathologist to check the actual lesion), giving the surgeon a sense of the patient’s status, and covering the result of preoperative imaging about the state of the tumor, all of which are important in the clinical evaluation of G.I. cases.

Precise comparison between the microscopic structure of the different types of G.I. tissue in benign and involved adjuvant or neoadjuvant states for each kind of G.I. organ is essential for future studies to clarify the relevance between impedance magnitude and IPS with the tissue structures.

So, our trial study on a calibrated EGCD system showed the autonomous capability of EGCD in real-time, detecting the complete or non-complete responses of dissected G.I. cancer tumors to chemo/radiation therapy and detecting the cancer nature of 61 cases with 120 detected lesions.

## 5. Conclusions

In this article, we designed and presented EGCD as a new complementary intraoperative diagnostic device that can be integrated with intraoperative frozen section pathology to detect G.I. cancer tumors. This is an observational study to present new calibration criteria for detecting G.I. cancers. The study aimed to better clarify the impact of real-time diagnostic warnings in informing surgeons about the presence or absence of malignant cells in dissected masses.

The measurements were carried out immediately after dissection on neoadjuvant and non-neoadjuvant tumors. This real-time EIS-based system is helpful for 3D scanning dissected G.I. tumors in either superficial or deep sites. Although our results covered all types of G.I. cancers, an individual trial for each kind of G.I. tumor may provide more clarification on the sensitivity and specificity of EGCD in each subtype of G.I. cancer. The data proved the reliability of EGCD scoring calibration in the intraoperative detection of G.I. cancer tumors.

The EGCD result could be helpful, especially in positive scores for surgeons, in giving insight into the status of the patients and covering the result of preoperative imaging about the state of the tumor. It could also help the pathologist evaluate the tumor, which is all-important in the clinical assessment of G.I. cases, making the EGCD invaluable.

## Figures and Tables

**Table 1:** Basic information on patients in the study cohort.

Characteristic	Category	Abundance (N = 61)	*p*-Value
Age	≥59 (Avg)	34 (55.7%)	0.383
	<59 (Avg)	27 (43.3%)	
Sex	Female	29 (47.5%)	0.264
	Male	32 (52.5%)	
Pathological Type	Adenocarcinoma	32 (52.5%)	_
	Squamous Cell Carcinoma (SCC)	17 (27.9%)	
	Neuro Endocrine Carcinoma	2 (3.3%)	
	Leiomyosarcoma	1 (1.6%)	
	Moderate Epithelial Dysplasia	1 (1.6%)	
	Signet Ring Cell Carcinoma (SRCC)	2 (3.3%)	
	Serous Carcinoma	2 (3.3%)	
	Tubulovillous Adenoma	3 (4.9%)	
	Mixed Adeno neuro endocrine Carcinoma (MANEC)	1 (1.6%)	
Presurgical Treatment	Chemoradiation therapy	48 (78.7%)	0.707
	Without/No known presurgical therapy	13 (21.3%)	
Organ	Esophagus	16 (26.2%)	_
	Gastric	22 (36.1%)	
	Colon	8 (13.1%)	
	Rectum	15 (24.6%)	

**Table 2:** Reporting the EGCD efficacy and comparison with other pre-post-surgical GIC evaluations.

			Presurgical Treatment	Post-Treatment Imaging Evaluation				Post-Surgical Pathology Evaluation		EGCD Score	
Sample	Total Number	Chemo/Radiation Therapy	Non-Chemo/Radiation Therapy	Viable Tumor	Non-Viable Tumor	Sensitivity	Specificity	Complete Response	Poor/No Response	Sensitivity	Specificity
Esophagus	30	30 (100%)	0 (0%)	6 (20%)	24 (80%)	81.3%	7.1%	16 (53.4%)	14 (46.6%)	81.3%	71.4%
Gastric	53	36 (68%)	17 (32%)	52 (98.1%)	1 (1.9%)	100%	20%	4 (7.5%)	49 (92.5%)	86.8%	73.3%
Colon	12	7 (46.6%)	5 (53.4%)	12 (100%)	0 (0%)	100%	0%	0 (0%)	12 (100%)	100%	100%
Rectum	25	21 (84%)	4 (16.6%)	23 (91.7%)	2 (8.3%)	93.7%	11.1%	6 (25%)	19 (75%)	81.2%	77.8%

## Data Availability

The datasets generated and analyzed during the current study are available from the corresponding author upon reasonable request.
